# Effect of N_2_/Ar Ratio on Wear Behavior of Multi-Element Nitride Coatings on AISI H13 Tool Steel

**DOI:** 10.3390/ma17194748

**Published:** 2024-09-27

**Authors:** Cheng-Hsun Hsu, Hong-Wei Chen, Chun-Yin Lin, Syue-Hong Hu

**Affiliations:** Department of Mechanical and Materials Engineering, Tatung University, Taipei City 10451, Taiwan; tonychen318@gmail.com (H.-W.C.); rupert475@gmail.com (C.-Y.L.); alex0979306954@gmail.com (S.-H.H.)

**Keywords:** H13 tool steel, cathodic arc deposition, N_2_/Ar flow ratio, (Ti, Cr, Cu, Al, Si)N coating, adhesion, wear resistance

## Abstract

In this study, multi-element nitride coatings composed of (Ti, Cr, Cu, Al, Si)N were synthesized on H13 tool steel using cathodic arc deposition (CAD) technology. The N_2_/Ar flow ratio varied from 0 to 2 as the experimental parameter, and two targets, Ti-Cr-Cu and Al-Si alloys, were utilized simultaneously. The impact of the gas flow ratio on the coatings’ abrasion properties was investigated, focusing on aspects, such as chemical composition, adhesion, hardness, and wear behavior. The experimental findings indicate that the coated specimens with a nitrogen reaction exhibit superior hardness and abrasion resistance compared to those without nitrogen use. While the surface roughness of the specimens tends to increase slightly with a higher N2/Ar ratio, the coating demonstrates improved hardness, adhesion, and abrasion resistance performance. In summary, the wear-resistant characteristics of H13 tool steel can be significantly enhanced when applying a CAD-(Ti, Cr, Cu, Al, Si)N film with a flow ratio of N_2_/Ar = 2.

## 1. Introduction

AISI H13 steel is widely recognized as a hot work tool steel, renowned for its composition comprising chromium (Cr), molybdenum (Mo), vanadium (V), and other alloying elements. This composition imbues H13 steel with exceptional toughness, abrasion resistance, and high-temperature fatigue resistance, rendering it suitable for prolonged usage in elevated temperature conditions. Additionally, its ability to withstand temperature fluctuations without significant deformation further underscores its utility. H13 steel finds extensive application in various industries, particularly in the fabrication of hot-forming molds, stamping molds, die-casting molds, and hot work tools [1,2,3]. However, due to cost considerations in the industry, the same die-casting mold or extrusion mold is usually used repeatedly. After a long period, the soldering phenomenon appears on the surface. This is the main reason for reducing the steel’s service life, so various surface treatments are often used to improve the tool steel’s service life. For example, Yaqoob et al. [4] used Al_2_O_3_/TiCN-CVD and TiAlN/AlCrN-PVD multilayer films to coat tool steel to improve the service life of cutting high-strength steel. Varis et al. [5] used heat treatment to enhance the wear resistance of HVAF and HVOF spray tool coatings. Poirier et al. [6] also used heat treatment to improve the ability of tool steel powder cold spraying. Temmler et al. [7] discussed the effect of laser micro polishing on the surface roughness of AISI D2 tool steel, and Ormanova et al. [8] used electron beam surface treatment to improve the hardness of tool steel to 718 kg/mm^2^.

The physical vapor deposition (PVD) method has been widely used in the field of industrial hard coatings in recent decades. The process of the PVD method is mainly carried out in a low-temperature vacuum reactor, using gas discharge to ionize the evaporated metal and the incoming gases (such as nitrogen, acetylene, etc.), as well as reacting to generate nitrides or carbides on the surface of the workpiece. Nitrides and carbides have very high hardness and generally exhibit strong adhesion, making them suitable for applications requiring wear resistance. In practical applications, there are many types of PVD-coated hard films, such as TiN [9,10], CrN [10,11,12], ZrN [13,14], CrAlN [15], TiCuN [16], TiAlN [17,18], and other binary/ternary nitrides [19,20,21,22] are mainly used in surface coatings of cutting tools and molds. They often have the advantages of high surface hardness and low friction coefficient.

Additionally, there is the possibility of applying a quaternary coating, such as a CrAlSiN composite ceramic film, which can be manufactured using PVD technology [23,24,25,26,27]. The related reports indicate that the film exhibits exceptional properties, including high hardness, excellent friction, and oxidation resistance. The wear resistance of CrAlSiN is superior to that of CrAlN, and the presence of silicon in the CrAlSiN film helps to lower its friction coefficient at room temperature. Chang et al. [26] used the cathodic arc vapor deposition method to coat TiAlN, CrAlSiN, and TiAlSiN films on tungsten carbide milling tools. The resulting TiAlN, CrAlSiN, and TiAlSiN had ultra-high hardness values of 31 ± 1 GPa, 36 ± 2 GPa, and 35 ± 2 GPa, respectively; the use life of CrAlSiN coated tools exceeded that of TiAlSiN- and TiAlN-coated tools [27]. This is because the CrAlSiN coating has a high wear resistance. Therefore, it can be seen that the nitride of multi-alloying element types is similar to that of high entropy alloys (HEAs) [28,29,30], which may have excellent properties as well as potential applications.

Therefore, this study employed the cathodic arc deposition (CAD) technique to coat H13 tool steel substrates with multi-elemental nitrides (Ti, Cr, Cu, Al, Si)N. The films were synthesized using co-reacting Ti-Cr-Cu and Al-Si dual targets, with variations in the N_2_/Ar gas flow ratio. Subsequently, the impact of the N_2_/Ar gas flow ratio on the wear resistance of H13 tool steel coated with this film was explored, aiming to enhance the longevity of the steel in service. Such research could provide a novel contribution to surface modification techniques for H13 tool steel.

## 2. Materials and Methods

### 2.1. Material Preparation and Pre-Coating Treatment

AISI H13 hot work tool steel substrates with a diameter of 25 mm and 6 mm thickness were used in this study, and their chemical composition was analyzed using glow discharge optical spectroscopy (GDOS), as listed in Table 1.

Before undergoing the coating procedure, the specimens were heated to form a high-hardness matrix suitable for practical applications. Specifically, a salt bath furnace was employed for this heat treatment process. The specimens were initially preheated at 550 °C for 15 min, and then further heated to 1050 °C for 30 min. Subsequently, they were quenched in water and tempered at 550 °C for 60 min, repeated twice. The outcome of the heat treatment revealed a significant increase in hardness, from the initial 13 HRC to 52 HRC.

It is a well-known fact that the roughness and cleanliness of the specimen’s surface play pivotal roles in determining the coating quality and adhesion. As such, the specimens underwent a series of preparatory steps including grinding, polishing, and cleaning before the coating process. The details were as follows: sandpaper was utilized to grind the surface of the specimens, followed by polishing using 0.3 μm aluminum oxide with deionized water until a mirror-like finish was achieved. Subsequently, the specimens were immersed in an acetone solution and subjected to ultrasonic shock for 10 min to ensure thorough cleaning. Upon completion of the cleaning process, the specimens were dried in an oven set at 70 °C for 15 min. This meticulous preparation ensured that the surfaces of all specimens were devoid of any contaminants, thus readying them for the coating process within the CAD chamber.

### 2.2. CAD Treatment

The coating process is outlined as follows: the specimens were positioned inside the CAD chamber, which was then evacuated to a high vacuum level of 5 × 10^−5^ torr. Subsequently, argon was introduced to regulate the pressure at 2 × 10^−2^ torr. A bias voltage of −700 V was applied, and argon ion bombardment was conducted for 10 min to further cleanse the specimens’ surfaces. Following the argon ion bombardment, the deposition process commenced. Initially, the Al-Si target was subjected to arcing for 5 min, resulting in the deposition of an AlSi intermediate layer on the H13 specimens. Subsequently, both the Ti-Cr-Cu and Al-Si targets were simultaneously activated, initiating the synthesis of films. To investigate the effect of N_2_ reactive gas, the N_2_/Ar flow ratios were manipulated as experimental parameters, specifically set at 0, 1, and 2, the three conditions in the study being numbered S0, S1, and S2 respectively. The coating process was concluded within 40 min, with the key process parameters listed in Table 2.

### 2.3. Coating Characteristics and Morphological Observation

In the study, various characteristics of the films were analyzed as follows. (1) The measurement of coating roughness was carried out with a surface roughness tester (Mitutoyo Surf test SV-400, Kawasaki, Japan) to obtain the average roughness (Ra value). (2) The chemical composition of the films was analyzed using a field emission electron probe micro-analyzer (EPMA, JEOL JXA-8530F Plus, Tokyo, Japan). (3) An X-ray diffractometer (Bruker D8 X-ray Powder Diffractometer, Karlsruhe, Germany) was used to identify the coating structure with Cu-target Kα radiation (λ = 1.54060 Å) at 40 kV and 30 mA, at a glancing incident angle of 2°; the scanning angle (2θ) ranged from 20° to 80° at a constant speed of 2°/min. (4) The adhesion strength quality (ASQ) of the coatings was evaluated by using Rockwell-C indentation testing with a load of 1471 N. The damage to the coatings was compared on a defined ASQ basis, where HF1–HF4 represented acceptable adhesion and HF5–HF6 represented insufficient adhesion (HF is the German short form for adhesion strength) [31]. (5) Nano-indentation measurements were performed using a nanoindenter (Hysitron TI 980 TriboIndenter, Hysitron, MN, USA) with a diamond Berkovich tip. According to the Oliver and McHargue method [32], both the values of hardness (H) and elastic modulus (E) for the coatings were measured by analyzing load–displacement curves under a load of 20 mN. The averages of 20 indentations for each film were reported with the standard deviation of both the H and E values. (6) A transmission electron microscope (TEM, Philips TECHNAIG-2F20, Cambridge, MA, USA) was used to observe multilayer morphology in the film, and a field emission scanning electron microscope (FE-SEM, HITACHI SU-8000, Tokyo, Japan) was used to measure the thickness of the coatings. (7) Also, a micro-hardness tester was used to measure the surface hardness of the coated specimens. A load of 300 g was applied in the micro-hardness tests. Each specimen was tested three times to obtain an average HV value.

### 2.4. Wear Test

**Figure 1:**
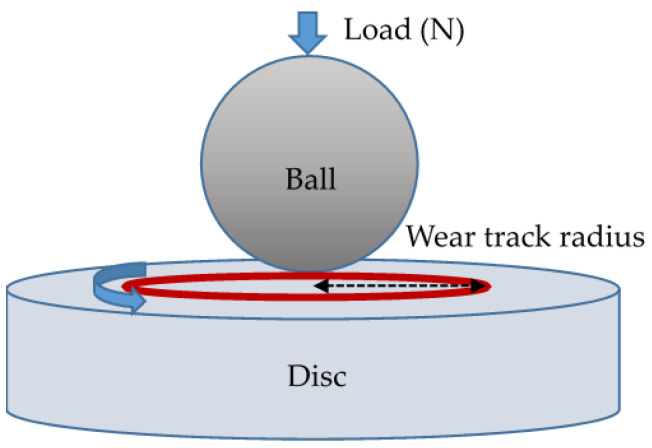
Schematic of the friction node used in the wear tests. The red circle is the wear track, and the dotted arrow is the wear track radius.

Based on previous wear test experiences [10,33], the wear tests were performed using a ball-on-disk tribometer (CSM Instruments, Peseux, Switzerland). The station view of the friction node used in the wear tests is shown in Figure 1. The testing conditions were as follows: (1) no lubricant, (2) circular track with a 5 mm radius against a 6 mm diameter WC-6%Co ball, (3) wear speed of 0.17 m/s, (4) a load of 2 N, (5) at room temperature, and (6) 35% relative humidity. The relationship between the friction coefficient and the total travel distance of 1200 m was continuously recorded during the testing. The wear rate was derived from the weight loss of the specimens, which was evaluated using a microbalance with an accuracy of 1 × 10^−4^ g. Three tests were performed for each coating condition, and the average wear rate was calculated. Furthermore, the worn surface of the specimens after the wear testing was observed using the FE-SEM.

## 3. Results and Discussion

### 3.1. Coating Composition and Structure

**Figure 2:**
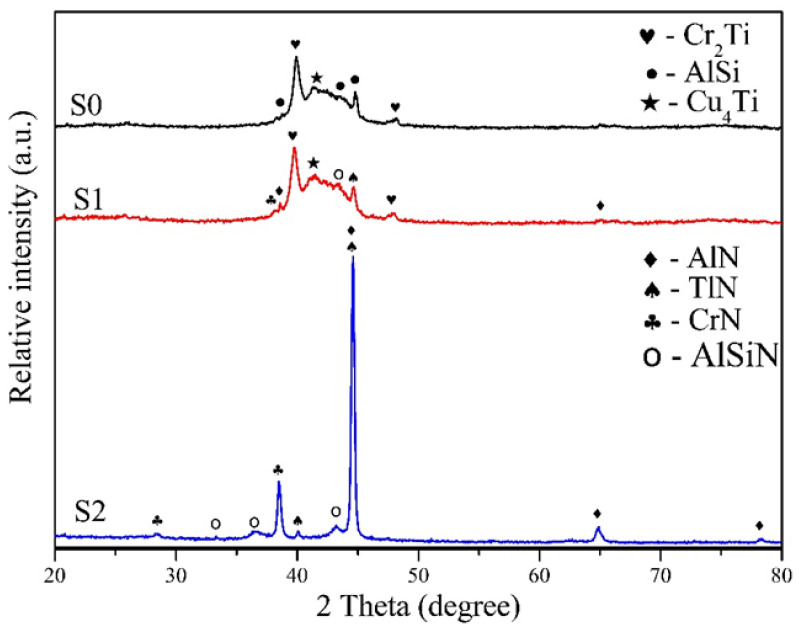
XRD patterns of the coatings in the study.

The resultant composition of the three coatings analyzed by EPMA is listed in Table 3, and the structure of the films was also examined using XRD, as shown in Figure 2. It can be found that the S0 film was synthesized without nitrogen addition, the content of Cu and Si was smaller as compared to Ti, Cr, and Al elements. The constitute phases in the coating are mainly composed of intermetallic compounds such as Cr_2_Ti, Cu_4_Ti, and AlSi. When nitrogen gas was added to the S1 specimen (N_2_/Ar = 1), the contents of Cu and Si in the coating were still low, and the contents of Ti and Cr were also reduced, while the content of Al was the highest. It could be found that the characteristic peaks of the XRD diffraction pattern appeared at 2θ = 44.7° for AlN(200), 2θ = 40.1° for TiN(110), and 2θ = 38.0° for CrN(111). Therefore, the coating structure of S1 contained a small amount of AlN, TiN, and CrN, attributed to the presence of nitrogen, alongside the formation of intermetallic compound phases, as observed in the S0 specimen. When the N_2_/Ar ratio reached 2 (S2), the nitrogen content in the coating increased significantly. As a result, nitrides such as AlN, CrN, and TiN became predominant in the coating structure, whereas the presence of intermetallic compounds was notably reduced. In addition, it can be seen that the AlN phase shows the strongest peak in the XRD pattern because of the highest content of Al and N in the S2 film (see Table 3). Due to the presence of a small amount of silicon, Si doping in AlN results in the formation of a minor quantity of AlSiN. The difference in the three coating structures may affect the abrasion properties of the coated specimens as discussed in the subsequent abrasion results.

### 3.2. Coating Morphology and Roughness

**Figure 3:**
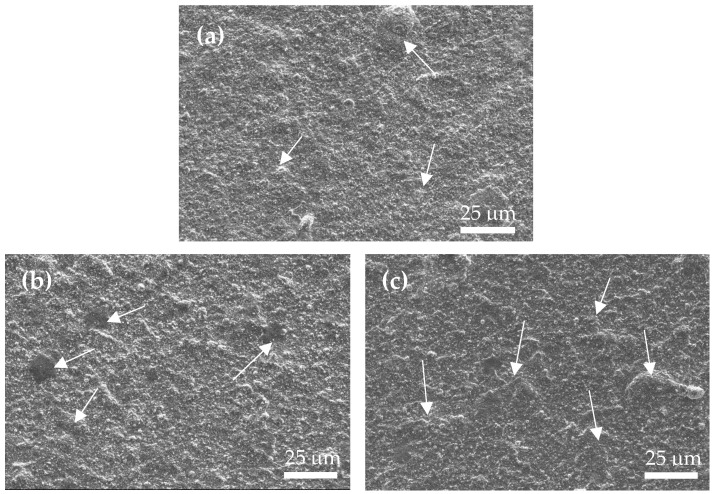
Surface morphology of the coated specimens. (**a**) S0, (**b**) S1, and (**c**) S2. The arrow points to the distribution of microdroplets.

**Figure 4:**
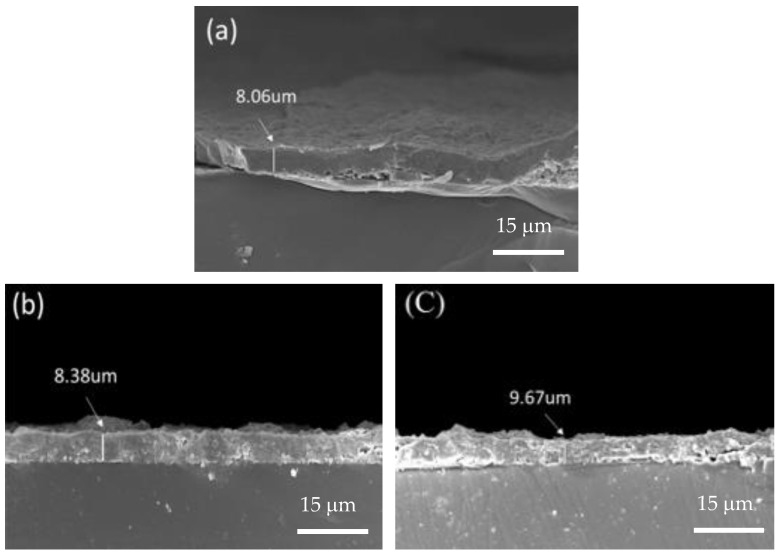
Cross-sectional view of the coated specimens: (**a**) S0, (**b**) S1, and (**c**) S2.

**Figure 5:**
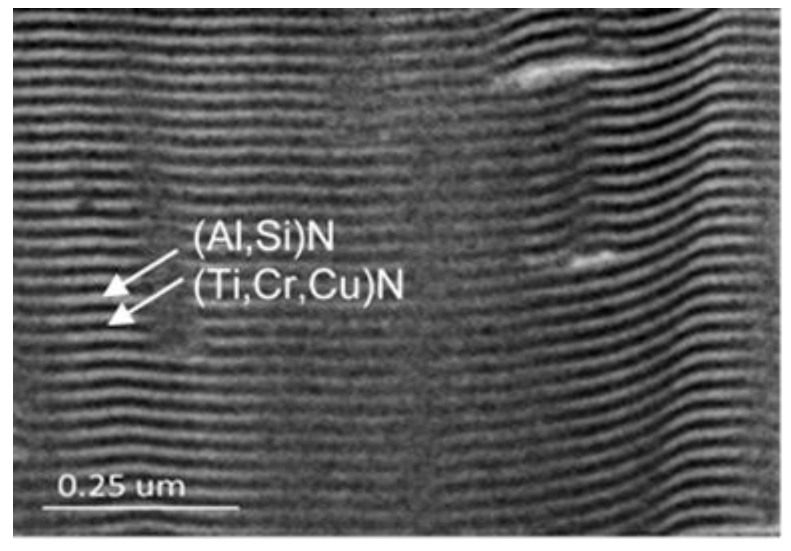
TEM cross-sectional appearance of the S2 multilayered film.

Figure 3 depicts the surface morphology of three coated specimens, revealing many micro-droplets on their surfaces. Figure 3a shows the S0 film without nitrogen, it seems that the size and quantity of the micro-droplets on the surface are smaller compared to the S1 and S2 films. Further comparing the difference between S1 and S2 (Figure 3b,c), it was also found that the droplet sizes on the S1 specimen seemed to be slightly smaller than those on the S2 specimen. To determine the coating thickness, the cross-sections of the coated specimens were examined using FE-SEM. Figure 4a–c show the cross-sectional morphologies of the three coated specimens, each with an average film thickness of approximately S0 = 8.06 μm, S1 = 8.38 μm, and S2 = 9.67 μm, respectively. This result also shows that the film thickness increases as the N_2_/Ar ratio increases. Figure 5 shows a high-magnification TEM cross-section of the S2 film. It can been seen there is a clear black and white nano-scale multilayered structure formed in the film. The reason is that the specimen carrier has a rotation rate of 4 rpm during the coating process. The white layer is approximately 17 nm thick and is (Al, Si)N, while the black layer about 10 nm thick and is (Ti, Cr, Cu)N. A greater thickness of the (Al, Si)N layer suggests that AlN forms more rapidly than the other nitrides at the same rotation speed, resulting in a thicker layer in the multilayer film. Moreover, the (Al, Si)N layer is composed of AlN mixed with a small amount of AlSiN phase.

**Figure 6:**
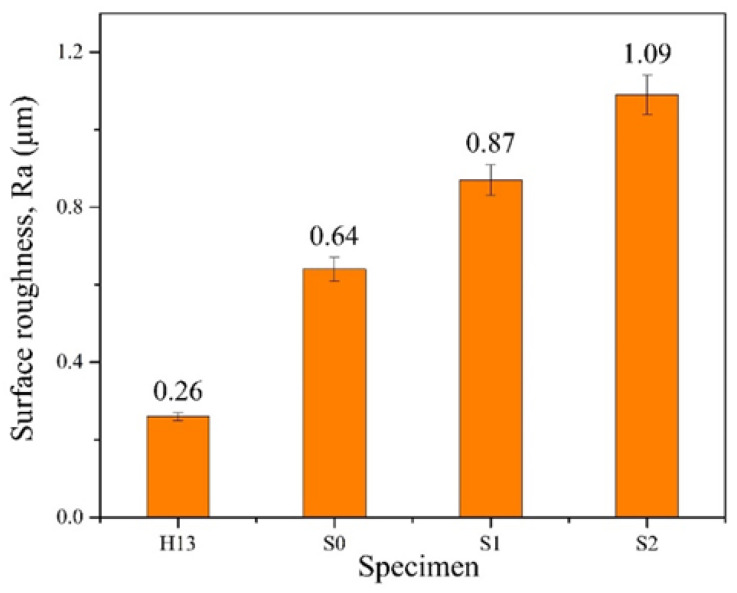
Surface roughness of the uncoated and coated specimens.

The surface roughness of the film layer is mainly affected by the sputtering of droplets on the surface of the coated specimen during the cathodic arc deposition process [34], i.e., the generation of droplets is due to the sputtering of larger neutral particles and atomic groups when the arcing position of the target is in the high-temperature melting state. In this study, the measurement of coating roughness was performed to obtain the average roughness (Ra value). The testing principle of surface roughness is to use a probe to scan the ups and downs of the surface of the object to make the probe vibrate vertically, and then collect the vertical vibration signal of the probe through a computer to achieve the measurement result. Because the Ra value is the average surface roughness, it is often used to represent the surface roughness of materials and is the most representative. Figure 6 illustrates the relationship between the surface roughness (Ra value) of each specimen before and after coating. The comparison reveals that the coated specimens exhibit higher Ra values, which can be attributed to microdroplets formed during the CAD process. Additionally, it is observed that the surface roughness increases with a higher N_2_/Ar ratio, resulting in S2 having a greater Ra value compared to S1.

### 3.3. Coating Adhesion and Hardness

**Figure 7:**
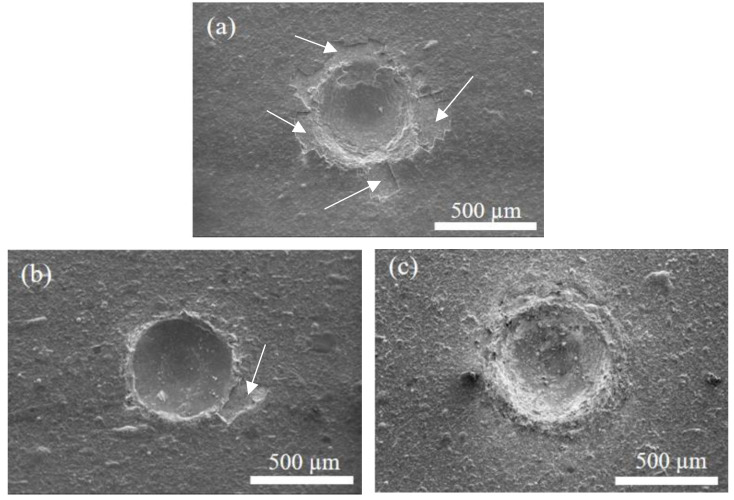
Adhesion comparison of the coated specimens. (**a**) S0, (**b**) S1, and (**c**) S2. The arrows point to the location where the film peels off.

In this study, the German specification VDI 3198 was used to determine the adhesion of the coating [31], i.e., an HRC indentation test of the film layer using Rockwell hardness machine, followed by observation of the rupture periphery of the film layer using SEM, and classification of the indentation in terms of adhesion according to the classification of rupture degree in the specification. Figure 7 shows the photographs of the indentation rupture on the surfaces of three coated specimens with arrows pointing to film peeling around the indentation. From Figure 7a, it can be found that the flaking of the S0 film without nitrogen is severe, and there is obvious film peeling around the indentation, so the S0 specimen can be assumed as HF4. Figure 7b is S1, and the graph shows that there is a little flaking around the indentation, but the extent of the flaking is lower than that of the S0 specimen, and therefore S1 is judged to be HF3. Figure 7c is S2, and this film almost has no obvious cracking and flaking. It has the best performance in terms of adhesion among the coated specimens; therefore, S2 can be classified as HF2. Therefore, the adhesion is S2 > S1 > S0 from the best to the worst among the three coated specimens.

**Figure 8:**
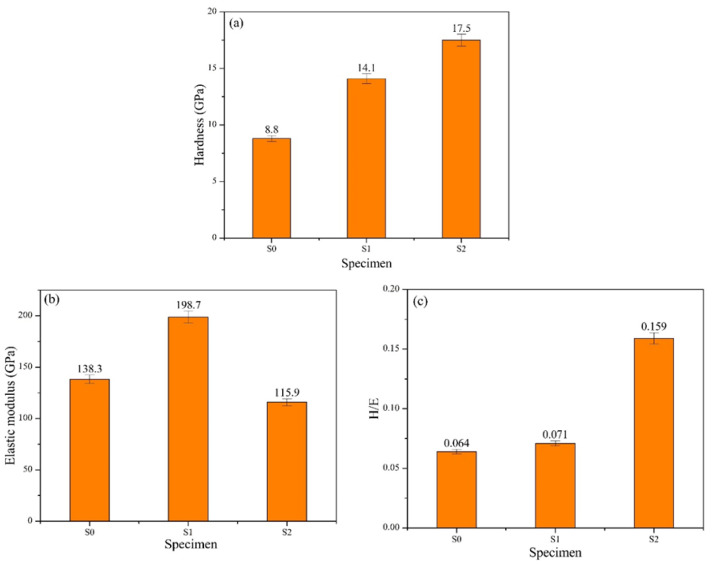
Comparison of (**a**) H, (**b**) E, and (**c**) H/E values for the coated specimens.

To understand the effect of N_2_/Ar flow ratio on film hardness and avoid the occurrence of substrate effect, this study used nano-indentation to accurately measure the film hardness (H) and its elastic modulus (E). Figure 8a–c shows the hardness (H), modulus of elasticity (E), and H/E values of the three types of coated specimens. The experimental results demonstrated that the hardness values were highest for S2 at 17.5 GPa, followed by S1 at 14.1 GPa, and lowest for S0 at 8.8 GPa. There is a notable difference in hardness between S0 and the other two specimens, S1 and S2. This disparity is attributed to the absence of nitride in the S0 film, resulting in its lower hardness. In contrast, both S1 and S2 films contain nitride, contributing to their higher hardness values. Particularly, the S2 film exhibited the highest hardness due to its greater nitride content compared to the S1 film. Regarding the H/E values, the elastic modulus (E) for the three coated specimens are as follows: S0 = 138.3 GPa, S1 = 198.7 GPa, and S2 = 115.9 GPa. Using these E values, the H/E ratios can be calculated. Comparing the H/E ratios of the three coatings, the ranking is S2 > S1 > S0.

**Figure 9:**
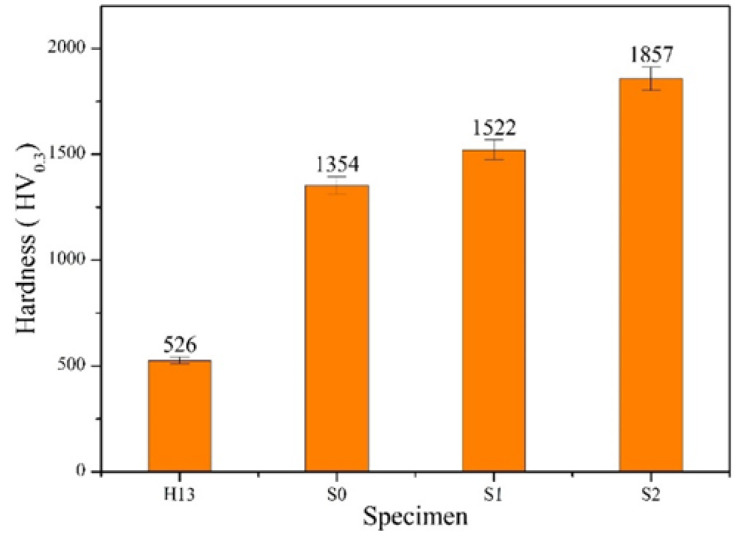
Comparison of Vickers hardness values among H13 and the coated specimens.

Additionally, Figure 9 illustrates a comparison of HV hardness values for the uncoated and coated specimens. The data indicate that the surface hardness of the uncoated H13 substrate, which measures 526 HV, can be substantially improved through coating. Among the coated specimens, S2 exhibits the highest hardness value at 1857 HV, followed by S1 at 1522 HV, and S0 with the lowest hardness value of 1354 HV. This hardness relationship ranking aligns with the previously discussed trend in nano-hardness variation (Figure 8a). Therefore, an increase in the amount of nitrides formed during coating markedly boosts the surface hardness of the H13 substrate.

### 3.4. Analysis of Wear Behavior

**Figure 10:**
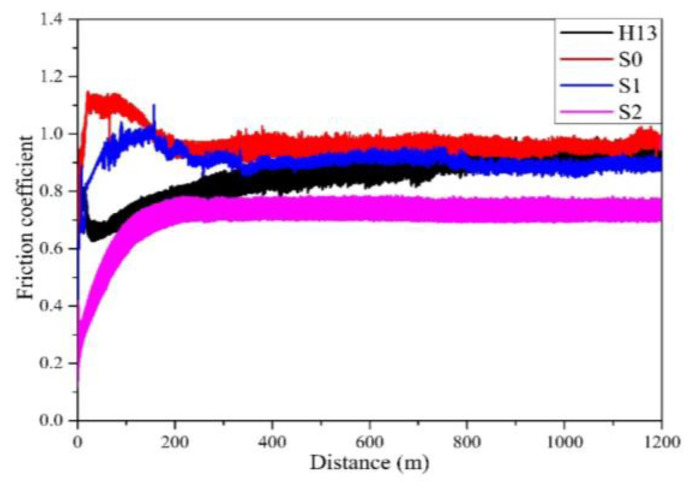
Friction coefficient curves of H13 and the coated specimens.

**Figure 11:**
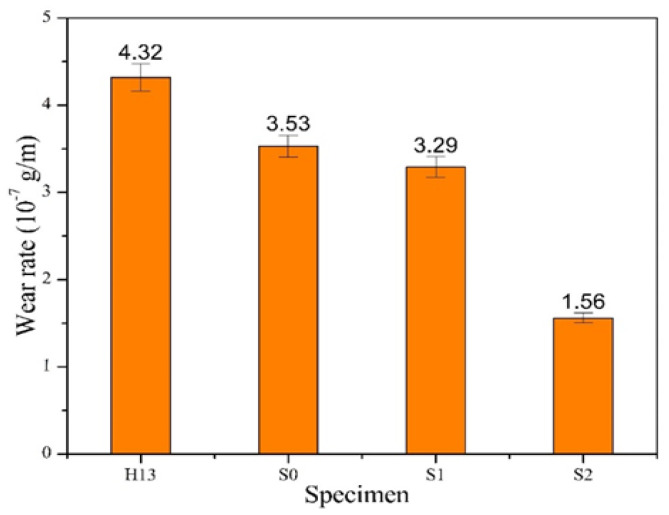
Comparison of wear rates among H13 and the coated specimens.

**Figure 12:**
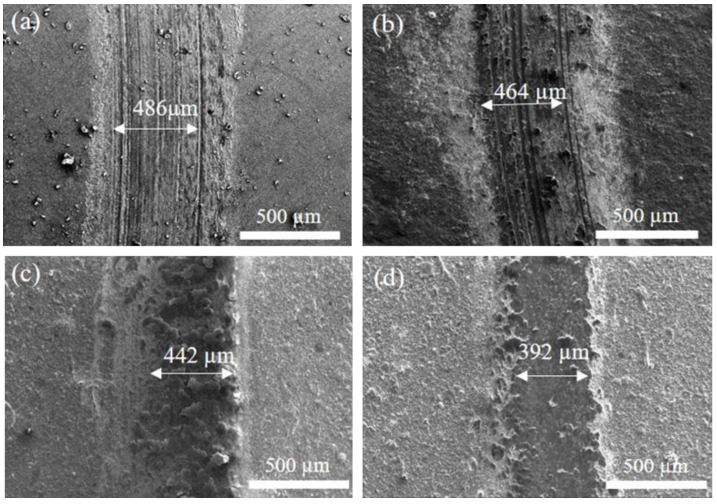
SEM surface morphologies of the uncoated and coated specimens after wear test: (**a**) H13, (**b**) S0, (**c**) S1, and (**d**) S2.

Some literature [35,36,37] pointed out that a higher H/E ratio in coatings is linked to reduced accumulated strain within the coating layer, thereby improving wear resistance. In other words, a higher H/E value is associated with better abrasion resistance. Therefore, the wear behavior of a coating material is affected by the H/E value. Figure 10 shows the correlation between the friction coefficient and the wear distance for H13 steel with and without coating after the 1200 m wear test. The curve in the figure shows that the friction coefficient of the uncoated H13 substrate seems to have a slightly rising trend, so its friction coefficient is slightly high. As a result, the uncoated substrate seems to have a higher friction coefficient among all the specimens. Further comparing the difference among the three coated specimens, we also find that after the wear distance of 800 m, the curves of friction coefficients for both the S0 and Si films are slightly close to each other to show a similar friction coefficient, while the S2 film has the lowest friction coefficient. This is because S2 has the most nitride content to show the highest hardness as well as the lowest friction coefficient. According to the curves in Figure 10, a comparison of the wear rate for each specimen can be further obtained, as shown in Figure 11. The comparison of data shows that the uncoated specimen has the highest wear rate, while the coating performance can reduce its wear rate. S2 has the lowest wear rate, so it has the best performance for improving the wear resistance. This result also matches those mentioned above, i.e., the abrasion behavior of the coating has a direct correlation with the H/E value. S2 has the highest H/E value to show optimal wear resistance. Figure 12 displays the SEM wear trajectory of the uncoated and coated specimens. From Figure 12a, it can be observed that the uncoated specimen after the wear test shows an apparent scratch and the wear width is the largest (486 μm), followed by the S0 and S1 specimens as seen in Figure 12b,c, which are 464 μm and 442 μm, respectively, while S2 has the narrowest wear width of 392 μm (Figure 12d). Therefore, it conforms to the comparison results in Figure 11, which show that S2 has the best wear resistance.

## 4. Conclusions

1. This study employed cathodic arc deposition to successfully coat (Ti, Cr, Cu)N and (Al, Si)N nano-scale interleaved multilayers on H13 steel, with good adhesion of the coating to the substrate.
2. In the absence of N_2_, the film structure consists primarily of Cr_2_Ti, Cu_4_Ti, and AlSi intermetallic compounds. When the N_2_/Ar ratio is set to 2, the film structure predominantly features AlN, TiN, and CrN nitrides, with a minor presence of AlSiN.
3. As the N_2_/Ar ratio increases, the hardness of the film rises, while the surface roughness of the coating also increases.
4. In terms of wear behavior, the wear resistance of H13 steel can be enhanced by applying a CAD-(Ti, Cr, Cu, Al, Si)N multilayer coating. The best wear resistance is achieved with a N_2_/Ar ratio of 2.

## Figures and Tables

**Table 1:** Chemical compositions of the H13 steel used in the study (wt.%).

Cr	Mo	V	Cu	C	Si	Mn	P	S	Fe
5.36	1.31	0.92	0.09	0.38	0.94	0.47	0.02	0.03	Bal.

**Table 2:** CAD processing parameters for the coatings in this study.

Parameter	Value
Two targets	(Ti34%-Cr34%-Cu32%) and (Al50%-Si50%)
N_2_/Ar flow ratio	(S0) N_2_(0 sccm)/Ar(180 sccm) = 0 (S1) N_2_(90 sccm)/Ar(90 sccm) = 1 (S2) N_2_(120 sccm)/Ar(60 sccm) = 2
Cathode current (A)	60
Working pressure (Torr)	$2\;\times \;10^{-2}$
Ar ion bombardment (V)	−700
Substrate bias (V)	−100
Substrate temperature (°C)	250
Rotation rate (rpm)	4
Distance between target and substrate (mm)	150
Total deposition time (min)	45

**Table 3:** Chemical composition of the films analyzed by EPMA (at.%).

Specimen	Ti	Cr	Cu	Al	Si	N
S0	26.25 ±1.31	32.46 ±1.66	8.54 ±0.42	27.99 ±1.39	4.76 ±0.23	0
S1	18.51 ±0.92	21.62 ±1.08	5.85 ±0.29	28.53 ±1.42	3.38 ±0.17	22.11 ±1.10
S2	11.26 ±0.56	10.41 ±0.52	3.02 ±0.15	28.77 ±0.14	3.42 ±0.17	43.12 ± 2.15

## Data Availability

The original contributions presented in the study are included in the article, further inquiries can be directed to the corresponding author.
